# Editorial: Understanding the link between lifestyle and neurodegenerative diseases

**DOI:** 10.3389/fnins.2024.1365734

**Published:** 2024-01-19

**Authors:** Yongting Wang

**Affiliations:** Med-X Research Institute, School of Biomedical Engineering, Shanghai Jiao Tong University, Shanghai, China

**Keywords:** neurodegenerative diseases, lifestyle, metabolism, physical exercise, APOE

In the intricate tapestry of neurodegenerative diseases, Alzheimer's stands as a formidable challenge, affecting millions globally. The quest to understand and mitigate its impact has led researchers to explore the dynamic interplay between lifestyle choices and the onset of Alzheimer's disease (AD). Among the multifaceted factors under scrutiny, the role of cholesterol profile and insulin resistance emerges as a crucial nexus.

In this series, we highlighted five papers to showcase the progress in the field. The work “*Physical exercise may increase plasma concentration of high-density lipoprotein-cholesterol in patients with Alzheimer's disease*” by Jensen et al., sheds light on a promising avenue in the realm of non-pharmacological interventions. This randomized controlled trial delves into the intricate relationship between physical exercise and lipid profiles in individuals grappling with AD. The foundation of this investigation lies in the recognition that lifestyle factors significantly influence the risk of developing Alzheimer's later in life. A cholesterol profile teetering toward the unfavorable and insulin resistance are identified as potent precursors to the onset of AD. Here, the spotlight turns to exercise, a modality that has shown cognitive benefits in healthy individuals. The paramount question addressed in this study: Can physical exercise wield a positive influence on the lipid profile, insulin, and glucose levels in patients with Alzheimer's?

The research, spanning 16 weeks, engaged 172 patients in a rigorous exploration of the effects of moderate-to-high intensity exercise. The cohort was divided into those embracing the exercise regimen and a control group undergoing treatment as usual. Intriguingly, the study considered the influence of apolipoproteinE genotype on key lipid components: total cholesterol, low-density lipoprotein-cholesterol (LDL-C), high-density lipoprotein-cholesterol (HDL-C), and triglycerides (TG) in plasma.

The findings resonate with a note of optimism. Significant attention was given to the “high exercise sub-group,” revealing a marked increase in plasma HDL-C levels. A 4.3% surge in HDL-C was observed in the high-exercise group compared to a marginal 0.7% decrease in the control group, after adjusting for statin use. These results, coupled with a stringent analysis of exercise adherence, propel the conclusion that short-term physical activity may indeed hold a key to enhancing the cholesterol profile in patients with AD.

The implications of this study extend beyond the immediate context, opening a door to interventions that embrace the vitality of lifestyle in our quest for healthier minds.

In the second installment of our series, we delve into a comprehensive review that underscores the interconnectedness of metabolic disorders and neurodegenerative diseases. Titled “*Animal models of metabolic disorders in the study of neurodegenerative diseases: an overview*” by de Bem et al., the authors navigate the complex landscape where obesity, diabetes, and hypercholesterolemia converge with Alzheimer's and Parkinson's disease.

Metabolic disorders are on the rise globally, mirroring the trajectory of neurodegenerative diseases. The review posits obesity, diabetes, and hypercholesterolemia as early harbingers of sporadic Alzheimer's and Parkinson's disease. Beyond the surface, these conditions share molecular and cellular signatures, including protein aggregation, oxidative stress, neuroinflammation, and blood-brain barrier dysfunction—hallmarks contributing to cognitive impairment and neuronal death.

The paper leverages rodent models of metabolic disorders as valuable tools for unraveling the phenotypic features and pathogenic mechanisms of neurodegenerative disorders. As we traverse the intricate terrain of animal models, we gain insights into the shared pathological aspects of Alzheimer's and Parkinson's disease.

The third paper, “*LPC-DHA/EPA-enriched diets increase brain DHA and modulate behavior in mice that express human APOE4*” by Scheinman et al., spotlights the intricate dance between APOE genotype and docosahexaenoic acid (DHA). APOE4, associated with heightened cognitive decline and increased risk of neurodegenerative disorders, prompts the quest for supplements targeting genotype-modulated processes. The study introduces a promising avenue—lysophosphatidylcholine (LPC)-DHA enriched diets, showcasing enhanced bioavailability and a potential shield against age-related neurodegeneration.

As we pivot to the fourth paper, “*Healthy lifestyles and wellbeing reduce neuroinflammation and prevent neurodegenerative and psychiatric disorders*” by Kip et al., the focus sharpens on the societal paradigm shift. From prioritizing productivity to emphasizing health and wellbeing, this paper asserts that embracing a balanced lifestyle is a win-win for individuals and societies. The integrated “healthy” lifestyle approach emerges as a cornerstone for preventing, rather than merely managing, neurodegenerative diseases.

The fifth and final paper, “*Liver's influence on the brain through the action of bile acids*” by Yeo et al., unveils the liver's pivotal role as a sensor and effector in peripheral metabolic changes. Aberrations in liver function, especially pertaining to bile acid composition, cast a far-reaching impact on the brain. The review explores the intricate interplay between liver dysfunction, bile acids, and neurological disorders, shedding light on an often-overlooked aspect of neurodegeneration.

Through this Research Topic, the threads connecting lifestyle choices to the intricate mechanisms of neurodegenerative diseases become more pronounced. From exercise-induced shifts in lipid profiles to the nuanced interaction between diet, genetics, and cognitive function, we navigate a landscape where lifestyle choices not only influence but might hold the key to mitigating the impact of neurodegenerative disorders.

## Author contributions

YW: Writing—original draft, Writing—review & editing.

